# A Contribution to the Prophylaxis of Lobar Pneumonia

**Published:** 1918-11

**Authors:** 


**A Contribution to the Prophylaxis of Lobar Pneumonia.** By John A. Kolmer and Edward Steinfield. *Journal of Infectious Diseases*, March, 1918.

Forty per cent. of healthy persons intimately associated with cases of lobar pneumonia, have been found harboring, in the mouth secretions, the same types of pneumococci as those producing the disease. Convalescents from pneumonia carry for a considerable time the type of pneumococcus with which they have been infected, as long as 90 days in one instance.

The authors have studied the problem of the destruction of pneumococci in the mouth secretions. They have tried this with solutions of various cinchonics in a menstruum of liquor thymolis, the latter solution possessing some germicidal activity for pneumoco cci and disguising the bitter taste of cinchona compounds.

In the experiments, both normal mouth secretions harboring Type IV pneumococci, and the sputum of pneumonia convalescents harboring Type I organisms were used. The pneumococcidal activity of the substance under experiments was determined by mouse noculation.

The complete destruction of all pneumococci in the mouth and upper air passages proved impossible with the agents used, but the systematic and daily use of washes prepared from 1 : 10,000 sol. of ethylhydrocuprein hydrochlorid, or quinin bisulphate, in 1/10 liquor thymolis, may serve efficiently to destroy virulent pneumococci as they gain access to the mucous membrane of the mouth and upper part of the the throat, and prevent their proliferation in large numbers. Ethylhydrocuprein hydrochlorid, by reason of its superior pneumococcidal activity, is to be preferred, but may be substituted by quinin bisulfate. Solutions of either, stronger than 1 : 10,000, may prove objectionable to most persons.

